# The effect of intervening hospitalizations on the benefit of structured physical activity in promoting independent mobility among community-living older persons: secondary analysis of a randomized controlled trial

**DOI:** 10.1186/s12916-017-0824-6

**Published:** 2017-03-28

**Authors:** Thomas M. Gill, Daniel P. Beavers, Jack M. Guralnik, Marco Pahor, Roger A. Fielding, Michelle Hauser, Todd M. Manini, Anthony P. Marsh, Mary M. McDermott, Anne B. Newman, Heather G. Allore, Michael E. Miller, for the LIFE Study Investigators

**Affiliations:** 1 Department of Medicine, Yale School of Medicine, Adler Geriatric Center, 874 Howard Avenue, New Haven, CT 06519 USA; 2 Department of Biostatistical Sciences, Wake Forest School of Medicine, Winston-Salem, NC USA; 3 Department of Epidemiology and Public Health, University of Maryland School of Medicine, Baltimore, MD USA; 4 Department of Aging and Geriatric Research, University of Florida, Gainesville, FL USA; 5 Tufts University, Nutrition, Exercise Physiology, and Sarcopenia Laboratory, Jean Mayer USDA Human Nutrition Research Center on Aging, Boston, MA USA; 6 Prevention Research Center, Stanford University School of Medicine, Stanford, CA USA; 7 Department of Health and Exercise Science, Wake Forest University, Winston-Salem, NC USA; 8 Departments of Internal Medicine and Preventive Medicine, Northwestern University Feinberg School of Medicine, Chicago, IL USA; 9 Department of Epidemiology, University of Pittsburgh School of Public Health, Pittsburgh, PA USA

**Keywords:** Mobility disability, Physical activity, Randomized controlled trial, Hospitalizations

## Abstract

**Background:**

Among older persons, disability is often precipitated by intervening illnesses and injuries leading to hospitalization. In the Lifestyle Interventions and Independence for Elders (LIFE) Study, a structured moderate-intensity physical activity program, compared with a health education program, was shown to significantly reduce the amount of time spent with major mobility disability (MMD) over the course of 3.5 years. We aimed to determine whether the benefit of the physical activity program in promoting independent mobility was diminished in the setting of intervening hospitalizations.

**Methods:**

We analyzed data from a single-blinded, parallel group randomized trial (ClinicalTrials.gov: NCT01072500). In this trial, 1635 sedentary persons, aged 70–89 years, who had functional limitations but were able to walk 400 m, were randomized from eight US centers between February 2010 and December 2013: 818 to physical activity (800 received intervention) and 817 to health education (805 received intervention). Intervening hospitalizations and MMD, defined as the inability to walk 400 m, were assessed every 6 months for up to 3.5 years.

**Results:**

For both the physical activity and health education groups, intervening hospitalizations were strongly associated with the initial onset of MMD and inversely associated with recovery from MMD, defined as a transition from initial MMD onset to no MMD. The benefit of the physical activity intervention did not differ significantly based on hospital exposure. For onset of MMD, the hazard ratios (HR) were 0.79 (95% confidence interval [CI] 0.58–1.1) and 0.77 (0.62–0.95) in the presence and absence of intervening hospitalizations, respectively (*P*-interaction, 0.903). For recovery of MMD, the magnitude of effect was modestly greater among participants who were hospitalized (HR 1.5, 95% CI 0.71–3.0) than in those who were not hospitalized (HR 1.2, 95% CI 0.88–1.7), but this difference did not achieve statistical significance (*P*-interaction, 0.670).

**Conclusions:**

Intervening hospitalizations had strong deleterious effects on the onset of MMD and recovery from MMD, but did not diminish the beneficial effect of the LIFE physical activity intervention in promoting independent mobility. To achieve sustained benefits over time, structured physical activity programs should be designed to accommodate acute illnesses and injuries leading to hospitalizations given their high frequency in older persons with functional limitations.

**Trial registration:**

ClinicalTrials.gov identifier NCT01072500.

**Electronic supplementary material:**

The online version of this article (doi:10.1186/s12916-017-0824-6) contains supplementary material, which is available to authorized users.

## Background

Prior observational research has shown that disability among older persons is often precipitated by intervening illnesses and injuries leading to hospitalization [1]. These intervening hospitalizations are associated with worsening functional ability for nearly all transitions between states of no disability, mild disability, severe disability, and death [2]. Strong evidence also exists that disability among older persons involves a complex interrelationship between baseline vulnerability and intervening events [3]. In the setting of an intervening hospitalization, disability is more likely to develop or worsen among persons who are highly vulnerable, with physical frailty (or functional limitations) being the most potent vulnerability factor. In an earlier observational study, the likelihood of developing long-term disability in mobility was increased more than 6-fold in the setting of an intervening hospitalization and 2.5- and 4.5-fold in the presence of moderate and severe functional limitations, respectively, as denoted by scores on the Short Physical Performance Battery (SPPB) [4].

In the Lifestyle Interventions and Independence for Elders (LIFE) Study, in comparison with a health education program, a structured moderate-intensity physical activity program significantly reduced the occurrence of a major mobility disability (MMD; hazard ratio [HR] 0.82), defined as the inability to walk 400 m, over an average follow-up of 2.6 years among 1635 sedentary persons aged 70–89 years who had functional limitations [5]. A subsequent analysis demonstrated that the physical activity program reduced the amount of time spent with MMD over the entire 3.5-year follow-up by 25% [6]. This reduction was accomplished not only by decreasing the initial occurrence of MMD, as shown in the earlier report [5], but also through enhanced recovery after an MMD episode and a diminished risk for subsequent MMD episodes. The LIFE physical activity intervention was not designed to prevent hospital admissions. In fact, participants who were randomized to the physical activity group were more likely to be hospitalized than those who were randomized to the health education group, although this difference did not achieve statistical significance [5].

Because disability is often precipitated by intervening hospitalizations and because these hospitalizations were observed more commonly in the physical activity group than the health education group, it is possible that the benefit of physical activity was diminished by intervening hospitalizations. The overall objective of the current analysis was to evaluate the effect of intervening hospitalizations on the benefit of structured physical activity in promoting independent mobility among older persons. We tested two related hypotheses: (1) intervening (i.e., incident) hospitalizations will be strongly associated with the initial onset of MMD and inversely associated with recovery from MMD among all participants, including those randomized to physical activity and those randomized to health education; and (2) the benefit of physical activity relative to health education on the onset of MMD and recovery from MMD will be diminished in the setting of intervening hospitalizations.

## Methods

### Trial design and participants

The LIFE Study was a multicenter, single-blinded randomized trial conducted at eight field centers across the USA between February 2010 and December 2013. Complete details of the methods have been published previously [7]. Men and women aged 70–89 years were eligible if they (1) were sedentary (reported <20 min/week in the past month of regular physical activity [i.e., exercise] and <125 min/week of moderate physical activity); (2) had functional limitations, as evidenced by a SPPB score ≤9 out of 12 [8]; (3) could walk 400 m in ≤15 min without help from another person or a walker; (4) had no major cognitive impairment (Modified Mini-Mental State Examination [9] [3MSE] 1.5 standard deviations below education- and race-specific normal values); and (5) could safely participate in the intervention as determined by their medical history, physical examination, and resting electrocardiography.

**Fig. 1:**
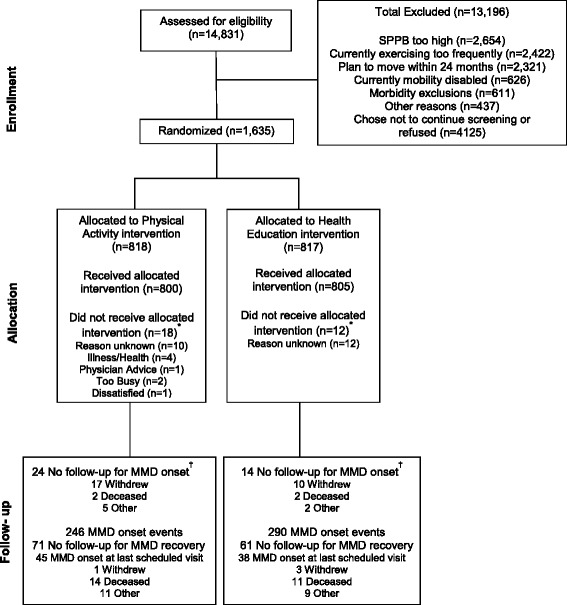
Flow of participants through the study. ^*^Participants who did not receive the allocated intervention (i.e., attended no intervention sessions). ^†^For participants who did not have any major mobility disability (*MMD*) assessments, we assigned 1 hour of follow-up time, because we knew that they were able to complete the 400-m walk at baseline. *SPPB* Short Physical Performance Battery

The primary recruitment strategy was targeted mass mailings to the community [10]. Additional strategies included newspaper, radio, and television advertisements and presentations at health fairs, senior centers, medical clinics, and churches. Eligibility was assessed sequentially, starting with a telephone interview, followed by a prescreening visit (at a subset of centers) and a first screening visit. During a second screening visit, eligibility was confirmed and participants were randomized (as described below). The flow of participants through the study is summarized in Fig. 1. The current manuscript presents results for a secondary analysis that was not pre-specified in the study protocol but was pre-specified in a proposal that was approved by the LIFE Publications and Presentations Committee prior to initiation.

The study protocol, available on request at https://www.thelifestudy.org/public/index.cfm, was approved by the institutional review boards at all participating sites. Written informed consent was obtained from all participants.

### Randomization

Participants were randomized to a physical activity or health education program via a secure web-based data management system using a permuted block algorithm (with random block lengths) stratified by field center and sex.

### Interventions

The two interventions, including safety, have been described in detail elsewhere [5, 7]. The physical activity intervention consisted of walking, with a goal of 150 min/week, strength, flexibility, and balance training [7]. The intervention included center-based sessions twice per week and home-based activity three to four times per week. Goals were individualized based on a participant’s level of physical fitness and were modified in response to illness, injury, or physical symptoms. The intensity of the center-based sessions was increased gradually, guided by the Borg scale of self-perceived exertion, which ranges in score from 6 to 20 [11]. Participants were asked to walk at an intensity of 13 (activity perception “somewhat hard”), and lower extremity strengthening exercises were performed at an intensity of 15–16. The behavioral strategies and operational details for implementing and maintaining the physical activity intervention over the course of 3.5 years are provided elsewhere [12]. When participants missed four or more consecutive sessions due to an intervening illness or injury, including hospitalization, they were placed on extended medical leave (as described below). On average, including extended medical leave, physical activity participants attended 63% of the scheduled sessions (median 71%, interquartile range [IQR] 50–83) [5]. Based on home logs, which were returned during each center-based session, participants reported walking a median of 81 min/wk (IQR 44–129) at home.

The health education group attended weekly workshops during the first 26 weeks and monthly sessions thereafter. Workshops covered topics of relevance to older persons, such as negotiating the healthcare system, traveling safely, and preventive services. The program also included a 5- to 10-min instructor-led program of upper extremity stretching exercises. There were no formal procedures for extended medical leave. On average, health education participants attended 73% of the scheduled sessions (median 82%, IQR 63–90) [5].

As previously reported [5], the physical activity intervention maintained a 104-min difference in walking and weight training activities compared with the health education group, based on the Community Healthy Activities Model Program for Seniors questionnaire [13], and a 40-min/wk difference in moderate physical activity assessed with accelerometry during the initial 2 years of follow-up, which included all participants.

#### Extended medical leave

A detailed protocol, described in the Manual of Procedures (available upon request at www.thelifestudy.org/public/index.cfm), was followed to manage extended medical leave. This protocol was considered an essential feature of the physical activity intervention. In brief, participants on leave were contacted every 2 weeks to obtain a status update, provide support, and assist them in making plans to resume the physical activity intervention, that is, return to the center-based sessions, when appropriate. The intervention staff were guided by state-of-the-art methods in phone-based “coach-oriented counseling” to facilitate resumption of physical activity. Illness was treated as an expected event in aging, and the message to participants was that their physical activity program had been put temporarily “on hold” until their medical condition had resolved. To ensure participant safety, approval from a health professional was required prior to restarting the intervention.

### Data collection

Participants returned to the clinic for follow-up every 6 months. The assessment staff was masked to the intervention assignment. Race and ethnicity were reported by the participants and were collected according to National Institutes of Health (NIH) requirements.

#### Ascertainment of intervening hospitalizations

Participants were asked about all hospital admissions since their last clinic visit. For each hospitalization, medical records were obtained and abstracted for diagnoses, procedures, and length of stay. During review of these records, the research staff and adjudicators identified additional hospitalizations (8.1% and 7.3% of all hospitalizations for the physical activity and health education groups, respectively) that had not been reported by participants. The reasons for each hospitalization were classified using the Medical Dictionary for Regulatory Activities (MedDRA®) scheme as previously described [14].

#### Outcomes assessment

The two outcomes included the initial onset of MMD and recovery from the first MMD episode. MMD was defined as the inability to complete a 400-m walk test within 15 mins without sitting and without the help of another person or a walker [7]. Use of a cane was acceptable. Participants were asked to walk 400 m at their usual pace, without overexerting, on a 20-m course for 10 laps (40 m/lap). Participants were allowed to stop for up to 1 min at a time for fatigue or related symptoms. When MMD could not be objectively measured because of the inability of the participant to come to the clinic and the absence of a suitable walking course at the participant’s home, institution, or hospital, an alternative adjudication of the outcome was based on the objective inability to walk 4 m in less than 10 s, or self-, proxy-, or medical record-reported inability to walk across a room [5]. If participants met these alternative criteria, they would not be able to complete the 400-m walk within 15 min. MMD was assessed or adjudicated during each follow-up visit through December 2013. Recovery was defined as a transition from initial MMD onset to no MMD [6]. Reports of death were tracked through regular surveillance.

### Statistical analysis

Sample size for the LIFE Study was based on the primary outcome of time until the initial onset of MMD, as previously described [5]. Baseline characteristics were summarized by study group using means (standard deviations [SD]) and percentages. For the onset of MMD, descriptive statistics were calculated by study group for three hospital exposures, including the percentage of participants hospitalized, number of hospitalizations, and number of days hospitalized. For recovery from MMD, only the percentages of participants with any hospitalization were calculated because the number of participants with more than one hospitalization during the at-risk period was small. The exposure periods for the two outcomes were from the time of randomization to initial onset of MMD (or censoring) and from the time of initial MMD onset to first recovery (or censoring). Observations were censored at the time of the last completed MMD assessment. These descriptive results were not compared statistically because a full evaluation comparing hospitalizations by study group has been previously published [14]. For descriptive purposes, the reasons for hospitalization were tabulated according to mobility outcome and study group.

Attendance at the center-based physical activity program was calculated separately for each 6-month interval, that is, the time between the follow-up visits, and these values were subsequently aggregated across the intervals. For the initial onset of MMD, values were calculated as the number of sessions attended divided by the number scheduled during the periods from: (1) no MMD to first hospitalization or next MMD assessment (or censoring) if no hospitalization occurred, and (2) first hospitalization to next MMD assessment (or censoring). For recovery from MMD, similar calculations were performed, except that the beginning of the period was defined by MMD (rather than no MMD). Intervals after MMD recovery were not included in these calculations. Attendance rates were estimated using a mixed linear model to account for differing numbers of potential intervention sessions per participant and the correlation between repeated attendance values within participants. Separate rates were estimated for participants at risk for MMD and recovery, and these analyses were rerun after omitting sessions during medical leave.

For both outcomes, time-to-event analyses were completed using Cox proportional hazards models. The independent variables included the presence/absence of an intervening hospitalization during the six months between MMD assessments as a time-varying covariate, study group, and their interaction. Contrast statements estimated the HR of each outcome by hospitalization status separately for the two study groups, as well as the treatment effect separately for participants who were hospitalized versus not hospitalized. Outcome rates were estimated using repeated measures Poisson regression models and generalized estimating equation methods. These models were fit using time-varying hospitalization status, study group, treatment by hospitalization interaction, and the two design variables (gender and clinical site).

For the onset of MMD, number of hospitalizations and number of days hospitalized prior to MMD within an assessment interval were evaluated as two alternate exposures. Number of days hospitalized was considered as a continuous variable, and HRs are presented for 0, 3 (median), and 10 days hospitalized. Recovery from MMD was evaluated among the subset of participants who developed incident MMD and had a subsequent MMD assessment. To reduce potential bias due to imbalances in baseline covariates or in attrition across study groups among participants with MMD, two additional models were run. The first used inverse probability weights to account for significant predictors of incident MMD, whereas the second used inverse probability weights to account for significant predictors of subsequent attrition (i.e., withdrawal/missed follow-up).

To determine whether the results differed significantly according to the severity of functional limitations, an interaction term denoting baseline SPPB subgroup (<8: moderate to severe versus 8–9: less severe) [10] was added to the final models. Because short-stay hospitalizations are less likely to be deleterious, the final models were re-run after omitting admissions of <2 days. All analyses were done using SAS, version 9.4 (SAS Institute, Cary, NC, USA). A Type I error rate of 0.05 and two-tailed alternative hypotheses were assumed for all comparisons.

## Results

As previously described [5], the baseline characteristics of participants in the two study groups were similar (Table 1). More than 40% had moderate to severe functional limitations, as denoted by an SPPB score <8. The median duration of follow-up was 2.7 years (IQR 2.3–3.1) and did not differ between the two groups. The median time to initial MMD onset was 2.5 years (IQR 1.9–3.0) in the physical activity group and 2.2 years (IQR 2.4–2.9) in the health education group. The corresponding values for MMD recovery were 0.5 years (IQR 0.4–1.3) and 0.6 years (IQR 0.4–1.5), respectively.

**Table 1:** Baseline characteristics of participants by study group

	Physical activity *N* = 818	Health education *N* = 817
Age in years, mean (SD)	78.7 (5.2)	79.1 (5.2)
Female sex, n (%)	547 (66.9)	551 (67.4)
Race/ethnicity, n (%)		
White, non-Hispanic	604 (73.8)	635 (77.7)
Black, non-Hispanic	163 (19.9)	125 (15.3)
Hispanic	31 (3.8)	30 (3.7)
Other	20 (2.4)	27 (3.3)
Education beyond high school, n (%)	544 (66.6)	550 (67.7)
Number of chronic conditions, mean (SD)	1.6 (1.1)	1.7 (1.0)
Hypertension, n (%)	573 (70.5)	578 (71.5)
Cardiovascular disease^a^, n (%)	236 (28.9)	254 (31.1)
Diabetes, n (%)	199 (24.4)	216 (26.6)
Cancer, n (%)	178 (21.9)	192 (23.6)
Chronic pulmonary disease, n (%)	130 (16.0)	123 (15.2)
3MSE score, mean (SD)	91.5 (5.5)	91.6 (5.3)
SPPB score		
Mean (SD)	7.4 (1.6)	7.3 (1.6)
<8, n (%)	353 (43.2)	378 (46.3)

*Abbreviations*: *3MSE* Modified Mini-Mental State Examination (0–100 scale), *SD* standard deviation, *SPPB* Short Physical Performance Battery

^a^Includes the presence of any of the following: self-report of a physician-diagnosed heart attack or myocardial infarction, stroke or brain hemorrhage accompanied by hospitalization, or heart failure; evidence of myocardial infarction based on a baseline electrocardiogram; or ankle brachial index ≤0.9 in either leg

Table 2 provides descriptive information on the hospital exposures by study group for participants at risk for the two mobility outcomes. For the initial onset of MMD, participants in the physical activity group were more likely than those in the health education group to be hospitalized, and they had a greater number of hospital admissions and days hospitalized. In contrast, for recovery from MMD, the proportions of participants who were hospitalized were comparable in the two study groups.

**Table 2:** Hospital exposures by study group according to mobility outcome

	Physical activity	Health education
Initial onset of MMD^a^		
Participants at risk, n	818	817
Participants hospitalized, n (%)	349 (42.9)	301 (36.8)
Time in years to first hospitalization, median (IQR)	1.0 (0.5–1.8)	1.1 (0.5–1.9)
Hospitalizations, n	611	501
Per participant, mean (SD)	0.8 (1.2)	0.6 (1.1)
Per participant hospitalized, mean (SD)	1.8 (1.2)	1.7 (1.2)
Days hospitalized, n	2679	2195
Per participant, mean (SD)	3.3 (7.4)	2.7 (6.3)
Per participant hospitalized, mean (SD)	7.7 (9.8)	7.3 (8.5)
Recovery from MMD^b^		
Participants at risk, n	246	290
Participants hospitalized, n (%)	72 (29.3)	87 (30.0)
Time in years to first hospitalization, median (IQR)	0.3 (0.1–0.6)	0.3 (0.2–0.8)

*Abbreviations*: *IQR* interquartile range, *MMD* major mobility disability, *SD* standard deviation

^a^From time of randomization to initial onset of MMD, which occurred in 246 (30.0%) and 290 (35.5%) of participants in the physical activity and health education groups

^b^From time of initial MMD onset to first recovery, which occurred in 89 (36.2%) and 98 (33.8%) of participants in the physical activity and health education groups

The proportion of short-stay admissions (i.e., <2 days) was similar for the two groups: 22.7% (physical activity) and 20.8% (health education) for initial onset of MMD and 15.4% (physical activity) and 17.0% (health education) for MMD recovery. The reasons for hospitalization are provided in Table 3 according to mobility outcome and study group. The most common reasons included cardiac disorders, nervous system disorders, surgical and medical procedures, and gastrointestinal disorders.

**Table 3:** Reasons for hospitalization according to mobility outcome and study group

	Initial onset of MMD		Recovery from MMD	
	Physical activity	Health education	Physical activity	Health education
Reason for hospitalization^a^	*N* = 611	*N* = 501	*N* = 246	*N* = 290
Blood and lymphatic system disorders	16 (2.6)	11 (2.2)	0 (0.0)	1 (0.3)
Cardiac disorders	86 (14.1)	72 (14.4)	28 (11.4)	19 (6.6)
Ear and labyrinth disorders	3 (0.5)	5 (1.0)	2 (0.8)	1 (0.3)
Endocrine disorders	5 (0.8)	1 (0.2)	0 (0.0)	0 (0.0)
Gastrointestinal disorders	66 (10.8)	41 (8.2)	15 (6.1)	10 (3.4)
General disorders and administration site conditions	21 (3.4)	13 (2.6)	2 (0.8)	6 (2.1)
Hepatobiliary disorders	8 (1.3)	5 (1.0)	0 (0.0)	1 (0.3)
Immune system disorders	6 (1.0)	2 (0.4)	1 (0.4)	1 (0.3)
Infections and infestations	58 (9.5)	42 (8.4)	12 (4.9)	12 (4.1)
Injury, poisoning, and procedural complications	40 (6.5)	43 (8.6)	17 (6.9)	15 (5.2)
Investigations	4 (0.7)	1 (0.2)	0 (0.0)	1 (0.3)
Metabolism and nutrition disorders	19 (3.1)	14 (2.8)	6 (2.4)	6 (2.1)
Musculoskeletal and connective tissue disorders	37 (6.1)	50 (10.0)	13 (5.3)	13 (4.5)
Neoplasms: benign, malignant, and unspecified^b^	13 (2.1)	14 (2.8)	4 (1.6)	4 (1.4)
Nervous system disorders	65 (10.6)	48 (9.6)	15 (6.1)	17 (5.9)
Psychiatric disorders	6 (1.0)	2 (0.4)	1 (0.4)	1 (0.3)
Renal and urinary disorders	11 (1.8)	11 (2.2)	4 (1.6)	3 (1.0)
Reproductive system and breast disorders	4 (0.7)	3 (0.6)	0 (0.0)	0 (0.0)
Respiratory, thoracic, and mediastinal disorders	47 (7.7)	35 (7.0)	13 (5.3)	5 (1.7)
Skin and subcutaneous tissue disorders	2 (0.3)	6 (1.2)	0 (0.0)	1 (0.3)
Surgical and medical procedures	65 (10.6)	64 (12.8)	10 (4.1)	16 (5.5)
Vascular disorders	29 (4.7)	18 (3.6)	6 (2.4)	8 (2.8)

Data are presented as n (%).

*Abbreviations: MMD* major mobility disability

^a^Based on MedDRA system Organ Class, listed alphabetically, as described in the Methods

^b^Includes cysts and polyps

During the course of the trial, a large proportion (481 of 818, 58.8%) of participants in the physical activity group were placed on medical leave at least once. A return to physical activity from medical leave was high after a hospitalization (681 of 817, 83.3%). Table 4 provides information on attendance at the center-based physical activity sessions before and after hospitalization for the two mobility outcomes. When medical leave was included, attendance at the center-based physical activity sessions was markedly lower after a hospitalization than before a hospitalization. After absences for medical leave were excluded, however, post-hospitalization attendance was only modestly lower than pre-hospitalization attendance. Regardless of medical leave, attendance was lower for MMD recovery than MMD onset.

**Table 4:** Attendance at the center-based physical activity sessions

	Onset of MMD (N = 818)		Recovery from MMD (N = 246)	
Medical leave	Before hospitalization	After hospitalization	Before hospitalization	After hospitalization
Included	61.4 (59.5–63.2)	23.9 (20.9–26.9)	28.5 (23.6–33.4)	8.3 (3.8–12.9)
Excluded	74.7 (73.2–76.1)	60.7 (56.7–64.5)	48.5 (42.4–54.6)	29.1 (16.7–41.4)

Data are presented as the mean percentage (95% confidence interval).

Attendance was calculated separately for each 6-month interval, i.e., the time between the follow-up visits, and these values were aggregated across the intervals as described in the Methods. For the onset of MMD, values were calculated as the number of sessions attended divided by the number scheduled during the periods from: (1) no MMD to first hospitalization or next MMD assessment (or censoring) if no hospitalization occurred, and (2) first hospitalization to next MMD assessment (or censoring). For recovery from MMD, similar calculations were performed, except that the beginning of the period was defined by MMD (rather than no MMD). Intervals after MMD recovery were not included in these calculations

*Abbreviations*: *CI* confidence interval, *MMD* major mobility disability

**Fig. 2:**
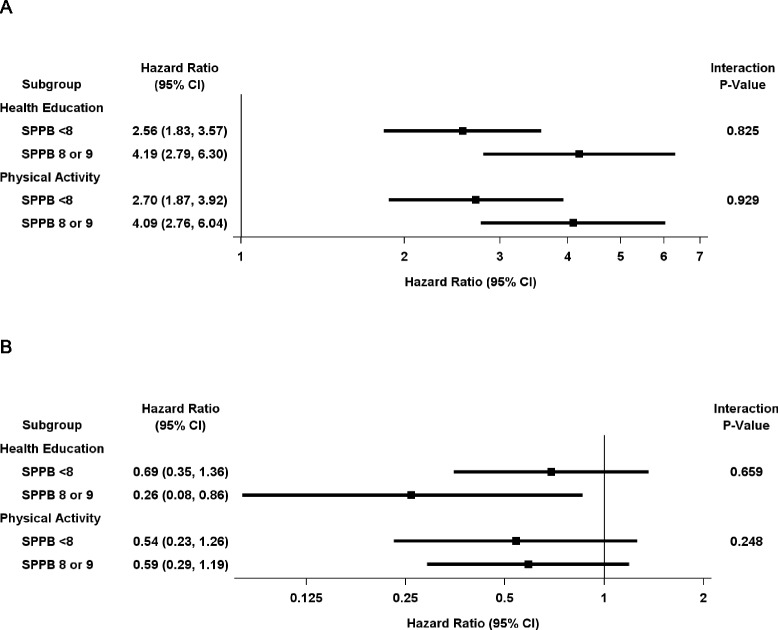
Associations between any hospitalization and (**a**) onset of major mobility disability (*MMD*) and (**b**) recovery from MMD according to study group and severity of functional limitations. *CI* confidence interval, *SPPB* Short Physical Performance Test

The associations between the hospital exposures and mobility outcomes are shown in Table 5. For both study groups, any intervening hospitalization was strongly associated with the onset of MMD, with nearly identical HRs of 3.3 (95% CI 2.5–4.3) and 3.2 (95% CI 2.5–4.1), representing more than a 3-fold elevation in risk. The results for number of hospitalizations and number of days hospitalized also showed no significant differences between the two study groups. In the setting of an intervening hospitalization, the likelihood of recovery from MMD was comparably diminished in both groups, with HRs varying from 0.59 (95% CI 0.39–0.88) to 0.60 (95% CI 0.35–1.04) for physical activity and from 0.45 (95% CI 0.27–0.74) to 0.51 (95% CI 0.29–0.92) for health education, depending on the specific multivariable model. These results did not differ significantly according to the severity of functional limitations, as shown in Fig. 2. After the short-stay admissions were excluded, the associations between the hospital exposures and mobility outcomes did not change substantively (Additional file 1: Table S1).

**Table 5:** Association between hospital exposures and mobility outcomes according to study group

	Physical activity (N = 818)			Health education (N = 817)			
	Outcome rate per 100 person-year			Outcome rate per 100 person-year			
Exposure^a^	Hospitalized	Not hospitalized	HR (95% CI)	Hospitalized	Not hospitalized	HR (95% CI)	*P* value^*^
	*Onset of MMD*						
Any hospitalization	39 (31–48)	10 (8–13)	3.3 (2.5–4.3)	46 (37–59)	14 (11–16)	3.2 (2.5–4.1)	0.903
Number of hospitalizations							
0 (ref)		10 (8–13)	1.0		14 (11–16)	1.0	0.742
1	36 (28–46)		3.0 (2.2–4.0)	39 (30–51)		2.7 (2.0–3.6)	
2 or more	50 (34–73)		4.3 (2.8–6.8)	79 (57–109)		5.1 (3.4–7.6)	
Number of days hospitalized^b^							
0 (ref)		13 (11–15)	1.0		16 (13–19)	1.0	0.804
3	15 (13–18)		1.2 (1.2–1.3)	19 (16–23)		1.2 (1.2–1.3)	
10	23 (19–28)		1.9 (1.6–2.1)	29 (24–36)		1.9 (1.6–2.2)	
Any hospitalization^c^	*Recovery from MMD* ^d^						
Model 1	43 (27–69)	64 (46–89)	0.60 (0.35–1.04)	33 (21–53)	51 (37–70)	0.51 (0.28–0.90)	0.670
Model 2	52 (33–80)	71 (50–99)	0.59 (0.39–0.88)	34 (21–54)	56 (40–77)	0.45 (0.27–0.74)	0.392
Model 3	43 (27–68)	64 (46–89)	0.60 (0.35–1.03)	33 (21–53)	51 (37–69)	0.51 (0.29–0.92)	0.688

*Abbreviations*: *CI* confidence interval, *HR* hazard ratio, *MMD* major mobility disability, *ref* reference group

^*^ Values represent statistical interaction between exposure and study group on mobility outcome

^a^Assessed during the interval preceding the outcome

^b^ Values for outcome rates are provided for a range of fixed values assuming a log-linear relationship. Three days was the median length of hospital stay, and 10 days allowed for long lengths of stay and more than one hospital admission

^c^ Results are not available for the number of hospitalizations or number of days hospitalized because the number of participants with more than one hospitalization during the at-risk period was small

^d^ All models include clinical site, age, and gender as covariates; Model 2 uses inverse probability weighting based on MMD, whereas Model 3 uses inverse probability weighting based on withdrawal/missed follow-up, as described in the Methods

**Fig. 3:**
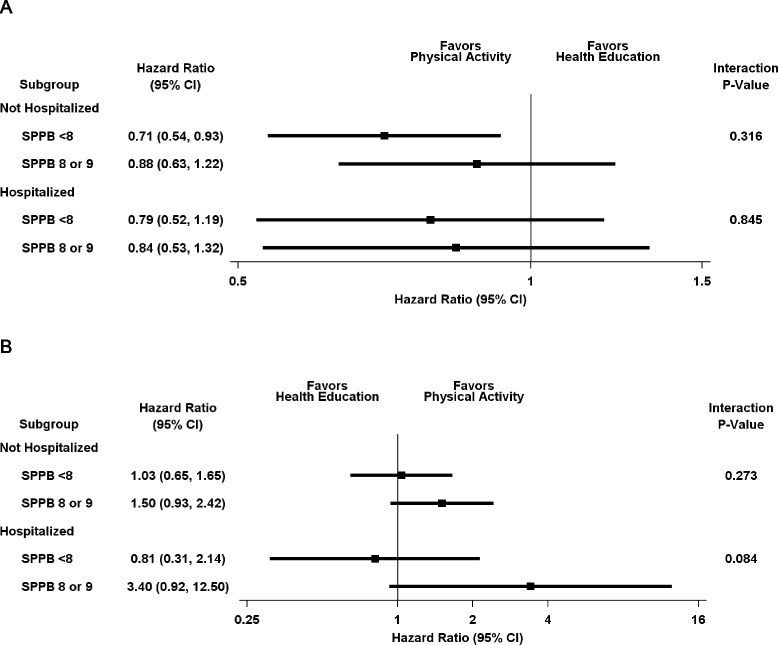
Effect of Physical Activity on (**a**) onset of major mobility disability (*MMD*) and (**b**) recovery from MMD according to hospital exposure and severity of functional limitations. *CI* confidence interval, *SPPB* Short Physical Performance Test

As shown in Table 6, the benefit of the physical activity intervention in promoting independent mobility did not differ significantly based on hospital exposure. For the onset of MMD, the hazard ratios (95% CI) were nearly identical in the presence or absence of a hospitalization. The results were similar for number of hospitalizations and number of days hospitalized, although the point estimates differed modestly. For recovery of MMD, the magnitude of effect was modestly greater among participants who were hospitalized than those who were not hospitalized, but the small samples sizes (shown in Table 2) led to relatively wide confidence intervals, particularly for the hospitalized group, and non-significant *P* values for each of the models. As shown in Fig. 3, the results for any hospitalization did not differ significantly according to the severity of functional limitations, although there was a suggestion of higher recovery in the setting of a hospitalization among participants in the physical activity group who had less severe functional limitations (*P* = 0.084). Exclusion of the short-stay hospital admissions had no meaningful effect on the benefit of the physical activity intervention (Additional file 1: Table S2). Among participants in the physical activity group, those who returned to the intervention after an intervening hospitalization had a lower annual rate of MMD onset (14.7% versus 79.4%) and higher annual rate of MMD recovery (52.0% versus 20.7%) than those who did not return to the intervention.

**Table 6:** Effect of physical activity on mobility outcomes within levels of hospital exposure

Exposure^a^	Operational definition	HR (95% CI)	*P* value^*^
	*Onset of MMD*		
Any hospitalization	Not hospitalized	0.77 (0.62–0.95)	0.903
	Hospitalized	0.79 (0.58–1.1)	
Number of hospitalizations	0	0.77 (0.62–0.95)	0.742
	1	0.85 (0.59–1.2)	
	2 or more	0.66 (0.37–1.2)	
Number of days hospitalized^b^	0	0.79 (0.66–0.95)	0.804
	3	0.80 (0.67–0.95)	
	10	0.81 (0.65–1.0)	
	*Recovery from MMD* ^d^		
Any hospitalization^c^			
Model 1	Not hospitalized	1.2 (0.88–1.7)	0.670
	Hospitalized	1.5 (0.71–3.0)	
Model 2	Not hospitalized	1.2 (0.94–1.6)	0.392
	Hospitalized	1.6 (0.90–2.9)	
Model 3	Not hospitalized	1.3 (0.88–2.1)	0.477
	Hospitalized	2.0 (0.71–5.4)	

*Abbreviations*: *CI* confidence interval, *HR* hazard ratio, *MMD* major mobility disability

^*^ Values represent statistical interaction between exposure and study group on mobility outcome

^a^ Assessed during the interval preceding the outcome

^b^ Results are provided for a range of fixed values. Three days was the median length of hospital stay, and 10 days allowed for long lengths of stay and more than one hospital admission

^c^ Results are not available for the number of hospitalizations or number of days hospitalized because the number of participants with more than one hospitalization during the at-risk period was small

^d^ All models include clinical site, age, and gender as covariates; Model 2 uses inverse probability weighting based on MMD, whereas Model 3 uses inverse probability weighting based on withdrawal/missed follow-up, as described in the Methods

## Discussion

In this secondary analysis of data from a large randomized clinical trial of community-living older persons, we found that intervening hospitalizations were strongly associated with the initial onset of MMD and inversely associated with recovery from MMD. However, they did not diminish the benefit of the physical activity intervention, relative to the health education intervention, in promoting independent mobility. These findings support the value of resuming physical activity after an illness or injury leading to hospitalization.

Given the established association between acute hospital admissions and adverse functional outcomes [1, 2, 4], which was confirmed in the current study, we had postulated that the benefit of physical activity in promoting independent mobility would be greater in the absence of an intervening hospitalization. Contrary to our expectations, the relative reduction in MMD onset was comparable among participants in the physical activity group who were hospitalized and those who were not hospitalized; these findings were consistent for three related, but distinct, hospital exposures.

In the setting of a hospitalization, the physical activity intervention may have reduced participants’ susceptibility to MMD by increasing reserve capacity or may have helped to facilitate recovery prior to their next MMD assessment. We have previously demonstrated that the physical activity intervention enhanced recovery after a documented episode of MMD [6]. In the current study, we found that the likelihood of recovery from MMD did not differ significantly between participants in the physical activity group who were hospitalized and those who were not hospitalized. These findings suggest that the strategies used to facilitate the return of participants to physical activity after an extended medical leave were effective in promoting recovery [12]. Indeed, we found that the rate of MMD recovery was considerably higher (and the rate of MMD onset was considerably lower) among participants who returned to the physical activity intervention after an intervening hospitalization than those who did not return. Because illnesses and injuries, including those leading to hospitalization, are so common among older persons [2], especially those with functional limitations (as shown in the current study), the long-term sustainability and success of a structured physical activity program will likely be highly dependent on such strategies.

The benefit of physical activity on reducing the onset of MMD was observed for hospitalized and non-hospitalized participants regardless of the level of functional limitations. For recovery from MMD, however, there was a suggestion of greater benefit in the setting of a hospitalization among participants in the physical activity group who had less severe functional limitations. Because these findings were based on <10% of the participants, they should be interpreted cautiously, but older persons with more severe functional limitations may not have sufficient reserve capacity, despite increasing their physical activity, to recover independent mobility after a disabling hospitalization.

The deleterious effects of the intervening hospitalizations on the two mobility outcomes (onset of MMD and recovery from MMD) were observed among all participants, including those randomized to physical activity and those randomized to health education; the magnitude of these associations did not differ significantly according to the severity of participants’ functional limitations. Although the role of intervening illnesses and injuries on the disabling process has been previously evaluated, prior research has been limited to observational studies [1, 2, 4, 15]. To our knowledge, this is the first clinical trial that has evaluated the effects of acute hospitalizations on functional outcomes or on the benefit of an intervention. When designing future trials in older persons, particularly trials focused on functional outcomes, investigators should anticipate and develop strategies to address the adverse consequences of intervening illnesses and injuries.

In the current study, the reasons for hospitalization were quite diverse, with no single reason leading to >15% of the hospitalizations. Given the small numbers, we chose not to evaluate the associations between the specific reasons for hospitalization and the two mobility outcomes. Information was not available on the severity of the hospitalizations, but our results did not change appreciably after short-stay hospital admissions, an indicator for low severity, were omitted.

Our study has other limitations. First, because the assessment intervals were every 6 months, some outcomes, that is, those lasting <6 months, could have been missed. Similarly, because it was not possible to ascertain the specific times for the onset of MMD or recovery from MMD, the temporal relationship between the acute hospitalizations and these outcomes cannot be firmly established. There is no reason to suspect that these limitations would bias comparisons between the two study groups, and prior research with assessment intervals of 1 month support the supposition that the acute hospitalizations likely preceded the mobility outcomes in most cases [2]. Second, because comparisons based on hospital exposure for MMD recovery occurred in non-randomized subsets of participants, there is no guarantee of balance on baseline characteristics between the two study groups. The results did not change appreciably in two additional models that used inverse probability weighting to account for potential bias. Finally, despite being the largest and longest randomized trial to evaluate the benefits of physical activity in older persons [7, 10], its power to detect small but meaningful treatment differences for subgroups defined on the basis of hospital exposure and severity of functional limitations was limited.

Study strengths include the large and racially diverse sample of vulnerable older persons from eight field centers spanning the USA, the long duration of the interventions and follow-up, excellent retention, and adherence rates to the physical activity intervention that were similar or higher than those achieved in other shorter studies involving older persons [16]. In addition, because <5% of age-eligible persons were excluded on the basis of an underlying medical condition, our results should be broadly applicable to our target population of sedentary older persons with functional limitations who do not already have MMD. Our assessment of MMD was based on an objective assessment of the ability to walk 400 m (about quarter of a mile), a distance that is required to carry out many activities and, hence, to be fully independent in the community [17]. Finally, rigorous procedures were used to ascertain and classify the acute hospital admissions, and our results for the onset of MMD were consistent across three different definitions of hospital exposure.

## Conclusions

The results of this secondary analysis of the LIFE Study suggest that hospitalizations had strong deleterious effects on the onset of MMD and recovery from MMD, but did not diminish the beneficial effect of the physical activity intervention in promoting independent mobility. To achieve sustained benefits over time, structured physical activity programs should be designed to accommodate acute illnesses and injuries leading to hospitalizations given their high frequency in older persons with functional limitations.

## Additional files

Additional file 1: **Table S1.** Association between modified hospital exposures and mobility outcomes according to study group. **Table S2.** Effect of physical activity on mobility outcomes within levels of hospital exposure. (DOCX 51 kb)

Additional file 2: Research investigators for the LIFE Study. (DOCX 49 kb)

## Abbreviations

3MSE: Modified Mini-Mental State Examination
CI: Confidence interval
HR: Hazard ratio
IQR: Interquartile range
LIFE: Lifestyle Interventions and Independence for Elders
MedDRA®: Medical Dictionary for Regulatory Activities
MMD: Major mobility disability
NIH: National Institutes of Health
SD: Standard deviation
SPPB: Short Physical Performance Battery
