# Supplementary material for: The effect of intervening hospitalizations on the benefit of structured physical activity in promoting independent mobility among community-living older persons: secondary analysis of a randomized controlled trial
Source: BMC Med. 2017 Mar 28;15:65. doi: 10.1186/s12916-017-0824-6 (PMC5368996; doi:10.1186/s12916-017-0824-6)
Supplement: Supplementary file 1 — Table S1. Association between modified hospital exposures and mobility outcomes according to study group. Table S2. Effect of physical activity on mobility outcomes within levels of hospital exposure. (DOCX 51 kb) [file 12916_2017_824_MOESM1_ESM.docx]

| **Table S1.** Association between Modified Hospital Exposures and Mobility Outcomes According to Study Group* | | | | | | | | |
| --- | --- | --- | --- | --- | --- | --- | --- | --- |
|  | Physical Activity | | |  | Health Education | | |  |
|  | Outcome Rate  per 100 Person-Year | |  |  | Outcome Rate per 100 Person-Year | |  |  |
| Exposure† | Hospitalized | Not  Hospitalized | Hazard Ratio (95% CI) |  | Hospitalized | Not  Hospitalized | Hazard Ratio (95% CI) | P- value‡ |
|  | *Onset of MMD* | | | | | | | |
| Any hospitalization | 46 (37, 58) | 11 (9, 13) | 3.9 (3.0, 5.1) |  | 49 (38, 63) | 14 (12, 17) | 3.2 (2.4, 4.2) | .289 |
| No. hospitalizations |  |  |  |  |  |  |  |  |
| 0 (ref) |  | 11 (9, 13) | 1.0 |  |  | 14 (12, 17) | 1.0 | .352 |
| 1 | 43 (33, 56) |  | 3.6 (2.7, 4.9) |  | 39 (29, 53) |  | 2.7 (1.9, 3.7) |  |
| 2 or more | 58 (39, 87) |  | 5.1 (3.1, 8.3) |  | 90 (64, 126) |  | 5.3 (3.4, 8.2) |  |
| No. days hospitalized§ |  |  |  |  |  |  |  |  |
| 0 (ref) |  | 13 (11, 15) | 1.0 |  |  | 16 (13, 19) | 1.0 | .815 |
| 3 | 15 (13, 18) |  | 1.2 (1.2, 1.3) |  | 19 (16, 23) |  | 1.2 (1.2, 1.3) |  |
| 10 | 23 (19, 28) |  | 1.9 (1.6, 2.1) |  | 29 (24, 36) |  | 1.9 (1.6, 2.2) |  |
| Any hospitalization\|\| | *Recovery from MMD*¶ | | | | | | | |
| Model 1 | 41 (25, 66) | 65 (47, 90) | 0.58 (0.33, 1.01) |  | 35 (21, 56) | 50 (37, 69) | 0.56 (0.31, 1.02) | .940 |
| Model 2 | 50 (32, 78) | 71 (50, 99) | 0.58 (0.39, 0.88) |  | 35 (22, 57) | 54 (39, 75) | 0.49 (0.29, 0.84) | .625 |
| Model 3 | 17 (9, 32) | 36 (23, 56) | 0.47 (0.22, 1.02) |  | 12 (6, 25) | 24 (16, 37) | 0.47 (0.20, 1.11) | .988 |
| Abbreviation: MMD, major mobility disability; CI, confidence interval; ref, reference group | | | | | | | | |
| *Hospital Exposures exclude admissions with length of stay less than 2 days. | | | | | | | | |
| †Assessed during the interval preceding the outcome. | | | | | | | | |
| ‡Values represent statistical interaction between exposure and study group on mobility outcome. | | | | | | | | |
| §Values for outcome rates are provided for a range of fixed values assuming a log-linear relationship. Three days was the median length of hospital stay, and 10 days allow for long lengths of stay and more than one hospital admission. | | | | | | | | |
| \|\|Results are not available for number of hospitalizations or number of days hospitalized because the number of participants with more than one hospitalization during the at-risk period was small. | | | | | | | | |
| ¶All Models include clinical site, age and gender as covariates; Model 2 uses inverse probability weighting based on major mobility disability, while Model 3 uses inverse probability weighting based on withdrawal/missed follow-up, as described in the Methods. | | | | | | | | |

| **Table S2.** Effect of Physical Activity on Mobility Outcomes Within Levels of Hospital Exposure* | | | |
| --- | --- | --- | --- |
| Exposure† | Operational Definition | Hazard Ratio (95% CI) | P-value‡ |
|  | *Onset of MMD* | | |
| Any hospitalization | Not Hospitalized | 0.74 (0.60, 0.91) | .289 |
|  | Hospitalized | 0.91 (0.66, 1.3) |  |
| No. hospitalizations | 0 | 0.74 (0.60, 0.91) | .352 |
|  | 1 | 1.0 (0.69, 1.5) |  |
|  | 2 or more | 0.71 (0.38, 1.3) |  |
| No. days hospitalized§ | 0 | 0.79 (0.66, 0.95) | .815 |
|  | 3 | 0.80 (0.67, 0.95) |  |
|  | 10 | 0.81 (0.65, 1.0) |  |
|  | *Recovery from MMD*\|\| | | |
| Any hospitalization¶ |  |  |  |
| Model 1 | Not Hospitalized | 1.3 (0.91, 1.8) | .940 |
|  | Hospitalized | 1.3 (0.62, 2.8) |  |
| Model 2 | Not Hospitalized | 1.3 (0.97, 1.7) | .625 |
|  | Hospitalized | 1.5 (0.81, 2.8) |  |
| Model 3 | Not Hospitalized | 1.4 (0.89, 2.3) | .988 |
|  | Hospitalized | 1.4 (0.50, 4.1) |  |
| Abbreviation: MMD, major mobility disability; CI, confidence interval | | | |
| *Hospital Exposures exclude admissions with length of stay less than 2 days. | | | |
| †Assessed during the interval preceding the outcome. | | | |
| ‡Values represent statistical interaction between exposure and study group on mobility outcome. | | | |
| §Results are provided for a range of fixed values. Three days was the median length of hospital stay, and 10 days allow for long lengths of stay and more than one hospital admission. | | | |
| \|\|Results are not available for number of hospitalizations or number of days hospitalized because the number of participants with more than one hospitalization during the at-risk period was small. | | | |
| ¶All Models include clinical site, age and gender as covariates; Model 2 uses inverse probability weighting based on major mobility disability, while Model 3 uses inverse probability weighting based on withdrawal/missed follow-up, as described in the Methods. | | | |
